# Testing Mechanical Properties of Rock Bolt under Different Supports Using Fiber Bragg Grating Technology

**DOI:** 10.3390/s19194098

**Published:** 2019-09-22

**Authors:** Xinxin Guo, Bo Wang, Zhenwang Ma, Zhenyu Wang

**Affiliations:** 1 Key Laboratory of Transportation Tunnel Engineering, Ministry of Education, Southwest Jiaotong University, Chengdu 610031, China; zj_gxinxin@my.swjtu.edu.cn (X.G.); mazhenwang7@my.swjtu.edu.cn (Z.M.); Wangzhenyu@my.swjtu.edu.cn (Z.W.); 2 School of Civil Engineering, Southwest Jiaotong University, Chengdu 610031, China

**Keywords:** fiber Bragg grating (FBG), prestressed anchor, non-prestressed anchor, loading mode, pullout test

## Abstract

Fiber Bragg grating (FBG) sensors, which can accurately measure strain, can be integrated with rock bolts with small fingerprints. In this paper, according to the force mechanism of prestressed anchor and non-prestressed anchor, different loading modes were designed, named active loading mode and passive loading mode. Then, FBG technology was used to monitor the axial force variation of prestressed anchor and non-prestressed anchor in different loading modes. Based on the test results, it is found that when the anchoring force is relatively small (<35 kN), prestressed anchors need to be tested by active loading mode, and non-prestressed anchors need to be tested by passive loading mode. For the prestressed anchor, the force condition of the bolt-shaft was similar to that of the two-force bar, and the axial force of the bolt-shaft was nearly the same along its entire length. Taking the applied load as the reference, the change rate of the axial force of the bolt-shaft was less than 10%. For non-prestressed anchor, due to the plate, there is a certain area surrounding the plate where the axial force of the bolt-shaft was greatly influenced. With applied loads of less than 15 kN, the change rate of the axial force on FBG1 was greater than 10%. With applied loads of greater than 20 kN, this was less than 10%. In this area, influenced by the plate, the axial force of the bolt-shaft increases, and as the applied load of the pullout test increases, the influence decreases.

## 1. Introduction

The rock bolt, one of the significant reinforcing members of the rock and soil mass, is widely used in the reinforcement and the support in hydraulic engineering, highway engineering, railway engineering, mining engineering, and port engineering, among others [1,2]. Two types of the rock bolts are mainly used in practical engineering: the non-prestressed anchor and the prestressed anchor. The non-prestressed anchor is a kind of passive supporting rock bolt. For example, the common cement grouted rock bolt, a representative of the fully grouted rock bolt, is the most popular type of the non-prestressed anchor. It is bonded firmly with the rock mass through colloidal cementitious materials (cement, epoxy resin, etc.) and reacts at the relative displacement between the rock bolt and the rock mass. Unlike the non-prestressed anchor, the prestressed anchor is a kind of active supporting rock bolt. Generally, the prestressed anchor tightens the nut or uses a loading system to implement the pre-load. It applies the load to the surface of the rock mass and transfers the tensile load to the internal rock mass.

The force condition of the rock bolt during the support process is a significant basis for assessing the anchorage performance. In practice, the pullout test is usually carried out on the rock bolt for the distribution of the axial force in order to assess the anchorage performance [3,4]. Compared with the loading process of the prestressed anchor, the loading method of the pullout test is extremely different from the load bearing mode of the non-prestressed anchor. As a result, the test data of the bolt-shaft axial force distribution may significantly vary from the actual one. Therefore, it is necessary to design the test process that is suitable in force mechanism for the accurate and reasonable axial force properties of the bolt-shaft in the laboratory test, and to further assess the loading performance of the prestressed anchor and non-prestressed anchor.

Up to now, conventional sensors such as the steel-wire sensor, vibrating-wire sensor, and strain gauges are usually used to measure the supporting effect of rock bolt. For example, the strain gauge and the steel string force-measuring rock bolt are used to understand the influence of the shape of bolt-shaft, the prestress, and the anchorage agent on the anchorage performance [5,6,7], the difference of the shaft force in various anchorage modes [8,9] and the failure mechanism of the rock bolt support [10]. Compared with the fiber Bragg grating (FBG) sensing technology that has been undergoing rapid development in recent years [11,12], traditional types of sensor, with low survival rate and poor durability, are vulnerable to moisture, difficult to connect in series, and unable to meet the requirements of intelligent testing [13]. Therefore, the application of optical fiber sensing test technology in rock bolt tests needs to be further explored.

As a fiber optical sensor, the FBG sensor has many advantages [14,15]. An FBG sensor has a simple structure, small size, and is lightweight, and it is suitable to be embedded in a structure to measure its strain and to detect the structural damage inside the structure [16]. An FBG sensor has excellent stability and repeatability with high sensitivity and high resolution. An FBG sensor is non-conducting and has minimum impact on the tested medium. An FBG sensor can realize semi-distributed sensing. In addition, with anticorrosion capacity and electromagnetic immunity, an FBG sensor is suitable for working in a harsh environment.

Using the advantages mentioned above, FBG sensors were first used to test the properties of composite materials in the aerospace industry and, nowadays, these sensors have found wide applications in biomedical engineering [17,18], civil engineering [19,20], composite structures [21], pipelines [22,23,24], and mechanical systems, among others. Mendez et al. [25] first introduced them to geotechnical testing. Currently, rock bolt is one popular geotechnical structure monitored using FBG sensors. Li et al. [26] analyzed the creep behavior of GFRP in concrete by embedding bare FBG sensors. Chai el al. [27] glued three FBG sensors to the groove of a bolt, and used them to record axial stresses and strains along the bolt during a pullout test. Weng el al. [28] adhered the FBG sensor to the surface of a tie-solder rod with “502” glue and wrapped with four layers of thermal plastic pipe, then monitored the seven strain conditions of the rock bolts during construction. Schroeck et al. [29] proposed a unique arrangement of FBG sensors to allow up to 20% strain measurements of steel rock bolts. Huang et al. [30] designed a novel distributed self-sensing fiber reinforced polymer (FRP) anchor rod with a built-in optical fiber sensor to predict the mechanical behavior of the anchor rod.

To accurately understand the mechanical properties of prestressed anchor and non-prestressed anchor, this paper uses FBG technology to design the loading mode during the test according to the stress characteristics of the prestressed anchor and the non-prestressed anchor. By testing and analyzing the mechanical properties of the rock bolt under different loading modes, the mechanical state changes of the rock bolt caused by different loading modes during the test are studied, and the reasonable loading mode of the prestressed anchor and non-prestressed anchor in the laboratory test is proposed.

## 2. FBG Sensor

### 2.1. Advantages of FBG in Bolt Monitoring

**Figure 1:**
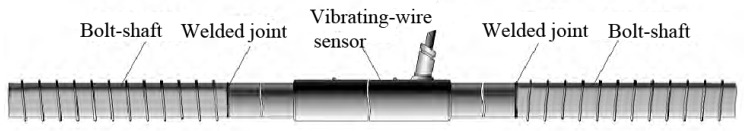
Installation diagram of the vibrating-wire sensor in bolt-shaft.

In order to obtain the stress state and anchorage mechanism of the rock bolt, various measurement techniques have been developed. Among them, the most commonly used traditional sensors are the vibrating-wire sensor and the electrical-resistance strain gauge [31]. The application of a vibrating-wire sensor in rock bolt generally needs to truncate the bolt-shaft, and then weld the sensor between the two bolt-shafts, as shown in Figure 1. Since the vibrating-wire sensors are generally large and the stress concentration exists in the welded joints, it is difficult to accurately test the force of rock bolt. The stability of resistance strain gauge is poor. The phenomenon of zero drift readily occurs in the process of using resistance strain gauges, and there is a sizeable residual strain in repeated measurements [32]. Moreover, some problems were encountered during in situ applications, including (i) corrosive groundwater and dusty circumstances causing failure of mechanical and electronic sensors, (ii) electronic sensors which were susceptible to electromagnetic interference, (iii) that monitoring of data collection relies on laborious manual operations, and (iv) implementation of real-time and remote monitoring was difficult [33].

The advantages associated with FBG sensors over conventional monitoring techniques include their immunity to electromagnetic interference; their small size and lightweight construction; and the access they facilitate to different measurements, such as strain, temperature, vibration, and specified chemicals. The FBG sensors can also be multiplexed, meaning that more than one sensor can be integrated along a single optical fiber. One can monitor the distributed mapping of a structure thanks to this multiplexing capability [34]. We finally chose the FBG sensor according to the requirements of this test of repeatability and accuracy.

### 2.2. Brief Introduction to the FBG Sensor

**Figure 2:**
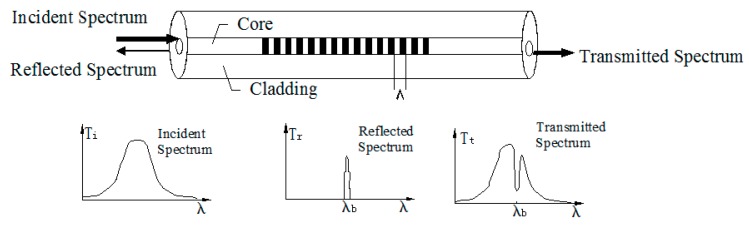
Working schematic of a fiber Bragg grating (FBG) sensor.

The basic working principle [35,36] of the FBG (Figure 2) is that when a light beam of wide spectrum enters the FBG, part of the spectrum produces an effective reflection under Bragg conditions. The peak wavelength of the reflected spectrum is called the Bragg wavelength $\lambda_{B}$, which satisfies the following equation:

(1) $$\lambda_{B}=2n_{eff}\Lambda ,$$

where $n_{eff}$ is the effective refractive index of the fiber core and Λ is the grating period (nm).

Any physical process that changes the above two parameters will cause a shift in the reflection and transmission wavelength. The change in the physical quantity (strain, displacement, pressure, acceleration, temperature, etc.) is obtained by determining the amount of shift of the wavelength:

(2) $$\Delta \lambda_{B}=\Delta \lambda_{\varepsilon }+\Delta \lambda_{t},$$

(3) $$\Delta \lambda_{\varepsilon }=K_{\varepsilon }\cdot \Delta_{\varepsilon }{,}_{\;}$$

(4) $$\Delta \lambda_{t}=K_{t}\cdot \Delta_{t}{,}_{\;}$$

where $\Delta \lambda_{B}$ is the variable quantity of the center wavelength of the grating; $\Delta \lambda_{\varepsilon }$, $\Delta \lambda_{t}$ are the variable quantities of the center wavelength of the grating caused by strain and temperature, respectively; $K_{\varepsilon }$ is the strain sensitivity coefficient; $\Delta_{\varepsilon }$ is the variable quantity of the strain of the member to be tested; $K_{t}$ is the temperature sensitivity coefficient; and $\Delta_{t}$ is the variable quantity in the temperature of the member to be tested.

### 2.3. FBG Sensor Installation

When the FBG sensor is used to measure the strain of the bolt-shaft, the conventional method is to first cut a slot on the bolt-shaft and then embed the FBG sensor in the groove [37,38]. The installation technology of the FBG sensor needs to ensure not only that the strain of the rock bolt can be fully characterized by the sensor, but also that the requirements of long-term monitoring should be met.

**Figure 3:**
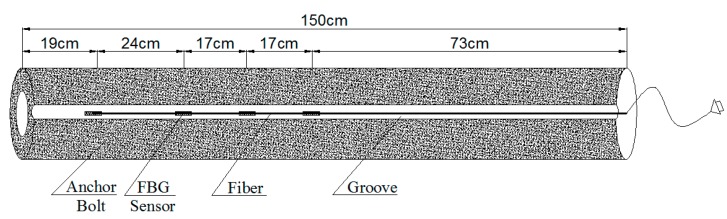
The concrete structure of the FBG force-measuring bolt-shaft.

**Figure 4:**
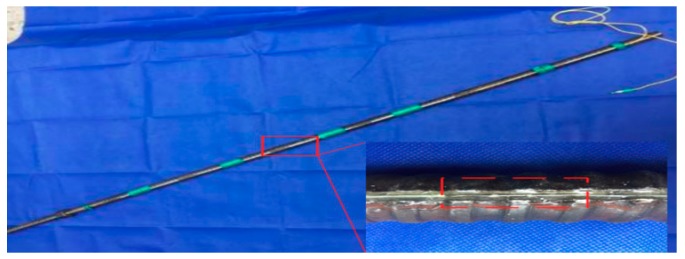
The post-package force-measuring bolt-shaft.

In the test, a rectangular groove, which is 4 mm in width and 3 mm in depth, is axially symmetrically extended along the axial direction of the bolt-shaft, and four FBG sensors are bonded in the groove by the adhesive. The FBG sensors are connected in series to a single fiber, and the fiber connector is drawn out at the end of the bolt-shaft. In order to meet the long-term stable monitoring requirements of the rock bolt, the FBG sensors are fixed to the 4 mm wide and 3 mm deep groove which is symmetrically extended along the axial direction of the shaft with adhesive material by a series of procedures as ‘bolt-shaft grooving→grinding machine polishing→groove cleaning→sensor pre-stretching→502 glue bonding→epoxy resin AB glue sealing→light pen inspecting’. The concrete structure of the FBG force-measuring bolt-shaft is shown in Figure 3, and the post-package force-measuring bolt-shaft is shown in Figure 4.

### 2.4. Data Interpretation

(1) Strain measure by FBG sensors

When the target load is reached, the FBG demodulator records a total of 4 s at 0.5 s intervals with 8 datasets per sensor. Among them, each time the first datum is used to trigger the signal, and, as a result, each sensor actually gets 7 datasets. According to these 7 datasets, an average wavelength is then obtained. Taking the first sensor as an example, the average wavelengths at 0 kN and 5 kN are respectively recorded as “$\lambda_{0}$” and “$\lambda_{1}$”, and the wavelength difference recorded as “$△\lambda_{1}$” is obtained by “$\lambda_{1\;}-{\;\lambda }_{0}$”. According to the calibration value of the above first sensor $k_{1\varepsilon }=1.1279\;\mathrm{pm}/\mu \varepsilon$, the strain value of the first sensor under 5 kN axial force can be calculated.

(2) Bolt-shaft strain

The bonding material between the packaged FBG sensor and the matrix material affects the strain transmission of the FBG sensor. The basic expression between the axial strain of the FBG sensor and the axial strain of the matrix material is given by [39]

(5) $$\varepsilon_{g}=\varepsilon_{m}\alpha \left(k,l\right),$$

where $\varepsilon_{m}$ is the axial strain of the rock bolt; $\varepsilon_{g}$ is the axial strain of the FBG sensor; and α(k,l) is the strain transfer coefficient and is defined as

(6) $$\alpha \left(k,l\right)=1-\frac{\sin h\left(kl\right)}{kl\cos h\left(kl\right)},$$

where l is the viscous length of the sensor and *k* is the parameter related to the characteristics of the fiber and the bonding material and can be solved by

(7) $$k^{2}=\frac{1}{\left(1+v_{c}\right)\frac{E_{g}}{E_{c}}r_{g\;}^{2}\ln\left(\frac{r_{c}}{r_{g}}\right)},$$

where $v_{c}$, $E_{c}$, $E_{g}$, $r_{g}$, $r_{c}$ represent the Poisson’s ratio of the bonding material, the modulus of elasticity of the bonding material, the modulus of elasticity of the fiber, the outer diameter of the fiber, and the outer diameter of the bonding material, respectively.

In this experiment, the Poisson’s ratio and elastic modulus of the adhesive material are 0.48 and 2.55 MPa, respectively. The elastic modulus of the fiber grating is 72 GPa, and the length of the FBG sensor is 20 mm. Parameter *k* can be calculated from Equation (7) as 58.933, and the strain conversion coefficient α(k,l) can be calculated from Equation (6) as 0.893.

**Figure 5:**
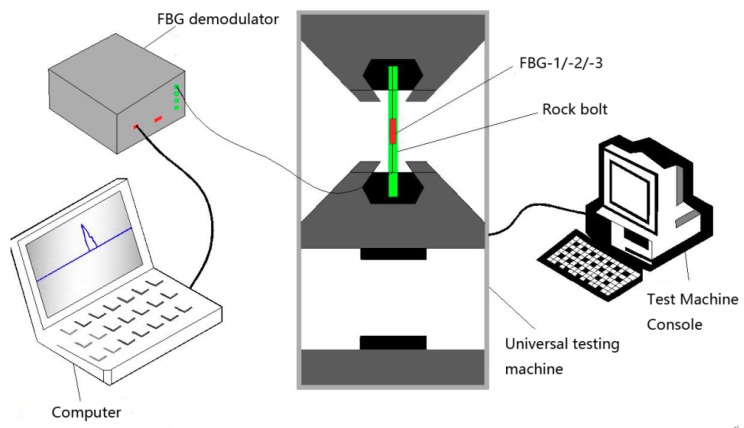
The calibration test for  α(k,l).

In addition, a calibration test for  α(k,l) is carried out, as shown in Figure 5. Three specimens were encapsulated in the same way. The length of the specimen is 50 cm with the FBG installed in the middle of the bolt where the force is equal to the applied one. The test load is from 0 to 40 kN, and the FBG sensors are recorded every 5 kN. Finally, conversion factors of FBG1, FBG2, and FBG3 are obtained, which are 0.903, 0.874, and 0.895, respectively. The average value is 0.891, which is very close to the theoretical calculation value. Finally, the conversion factor is chosen as 0.893, with the error ranging from −0.019 to 0.01 and the error rate below 2.1%.

(3) Axial force of bolt-shaft

The relationship between the axial force of bolt-shaft and bolt-shaft strain is given by

(8) $$F=A\sigma_{m},$$

where F is the axial force of bolt-shaft, A is the cross-section area of bolt-shaft, and $\sigma_{m}$ is the bolt-shaft stress and can be solved by

(9) $$\sigma_{m}=E\varepsilon_{m},$$

where E is the elastic modulus of bolt-shaft.

When the wavelength of the FBG sensor is offset to 1 pm, the axial force of bolt-shaft can be solved by

(10) $$F=\frac{EA}{K_{\varepsilon }\alpha \left(k,l\right)},$$

such that the conversion coefficient between the wavelength offset measured by FBG sensor and the axial force of bolt-shaft can be defined as β, and the value is $\frac{EA}{K_{\varepsilon }\alpha \left(k,l\right)}\;N/\mathrm{pm}$. For the conversion coefficient β, the error comes from E, $K_{\varepsilon }$, and α(k,l). When the bolt-shaft material is low-carbon steel, the elastic modulus E is 201 ± 5 GPa with the change rate of 2.5%. $K_{\varepsilon }$ is given by the calibration test. The calibration curve shows that the linear correlation is greater than 0.99, and the change rate is less than 1.0%. For a change rate of α(k,l), it is less than 2.1%. Hence, the change rate of β is less than 5.7% = (1 + 2.5%) × (1 + 1%) × (1 + 2.1%) − 1.

## 3. Rock Bolt Mechanism and Experiment Condition Design

### 3.1. Action Mechanism of the Prestressed Anchor

The prestressed anchor, which provides active support, can transfer the tensile load to the internal rock mass by tightening the nut or by using a loading system. The bolt-shaft can be divided into the fixed length and the free length. The force acting on the prestressed anchor comes from two parts: the force in the fixed length, and the applied load.

**Figure 6:**
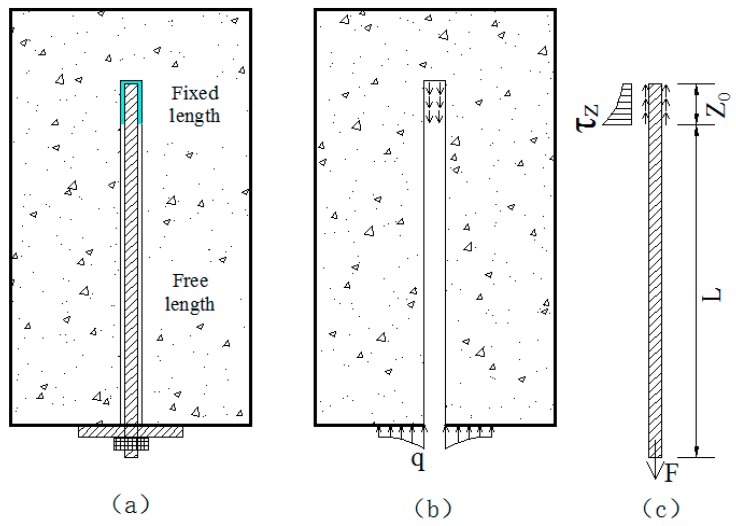
Mechanism of the prestressed anchor. (**a**) the schematic of “rock bolt—surrounding rock” supporting structure, (**b**) the force schematic of the surrounding rock, (**c**) the force schematic of the bolt-shaft.

According to what is mentioned above, the mechanical model of “rock bolt—surrounding rock” can be established, as shown in Figure 6. Figure 6a is the schematic of “rock bolt—surrounding rock” supporting structure, Figure 6b is the force schematic of the surrounding rock and Figure 6c is the force schematic of the bolt-shaft.

### 3.2. Mechanism of the Non-Prestressed Anchor

The non-prestressing anchor is a composite anchorage system, as depicted in Figure 5. For example, the cement grouted rock bolt, whose body is bonded firmly with the rock mass through colloidal cementitious materials (cement, epoxy resin, etc.), is a representative one. The mechanism of the non-prestressed anchor is that the rock bolt and surrounding rock must produce relative deformation first, then the rock bolt can provide the supporting force. If there is no relative deformation, the supporting force will not be produced. In other words, it is not until there is relative deformation between the rock bolt and the surrounding rock that the rock bolt starts to work. As a consequence, the end of the rock bolt will have a quite large displacement when the rock bolt reaches its full bearing capacity. Furthermore, this large displacement may even exceed the allowable limit of the rock (foundation) or the structure. Therefore, the supporting effect of this kind of rock bolt to surrounding rock is mainly exerted by the anchor plate and the anchorage body. The force acting on the non-prestressed anchor also comes from two parts: the force between the fixed length of bolt-shaft and the anchorage body, and the force between the anchor plate and the surrounding rock.

**Figure 7:**
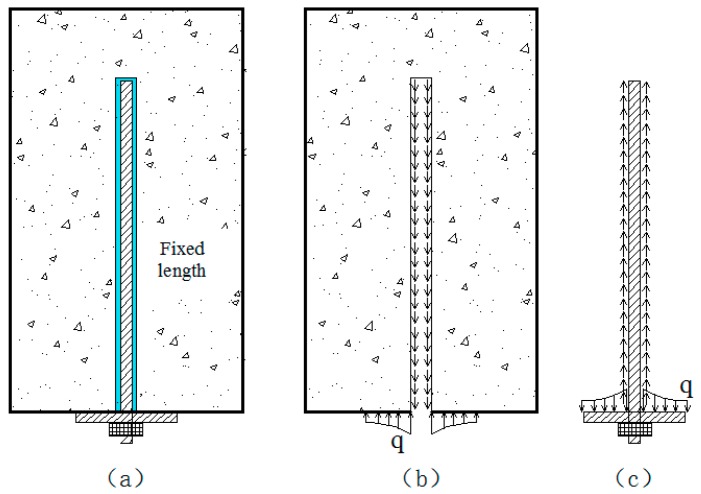
Mechanism of the non-prestressed anchor. (**a**) the schematic of “rock bolt − anchorage body − surrounding rock” supporting structure, (**b**) the force schematic of surrounding rock, (**c**) the force schematic of the rock bolt.

According to what is mentioned above, the mechanical model of “rock bolt − anchorage body − surrounding rock” can be established, as shown in Figure 7. Figure 7a is the schematic of “rock bolt − anchorage body − surrounding rock” supporting structure, Figure 7b is the force schematic of the surrounding rock, and Figure 7c is the force schematic of the rock bolt.

### 3.3. Design of Experiment Condition

The primary purpose of this experiment is to propose a reasonable loading mode of the prestressed anchor and the non-prestressed anchor in the laboratory test. Therefore, in the designed experiment condition, the borehole is not grouted. In addition, a jack was placed onto the concrete specimen to ensure that the rock bolt was in a simple tension state. In this way, the influence of the bending moment of the bolt body during the tensile process can be eliminated. Based on the stress characteristics of the prestressed anchor and the non-prestressed anchor obtained from the analysis of their mechanisms in Section 3.1 and Section 3.2, two groups of experimental conditions were designed.

**Figure 8:**
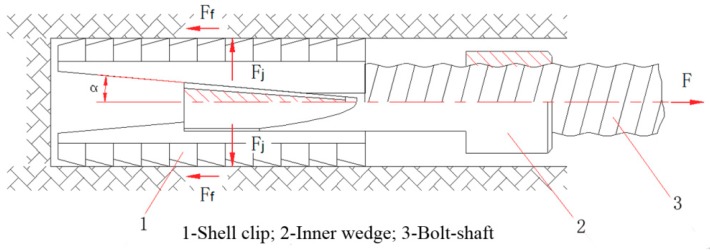
Structure and principle of expansion shell.

The mechanical anchoring (expansion shell) was adopted to simulate prestressed anchor. The structure and principle of expansion shell are shown in Figure 8. When a load *F* is applied to the rock bolt, the bolt-shaft is loaded, and the inner wedge is moved outward, and after that, an extrusion force F_j_ is applied to the shell clip to press the shell clip against the wall of the borehole tightly. Then, a friction resistance F_f_, opposite to the load direction of the bolt-shaft, is generated to balance the force of the supporting system and prevent the continuous movement of the expansion shell. The force characteristic of the mentioned expansion shell makes it possible to apply prestress to the bolt-shaft. The frictional resistance F_f_ between the wedge and the wall of borehole increases as the load *F* applied to the bolt-shaft increases. Therefore, the anchoring system can be locked in the borehole under a certain applied load *F*.

**Figure 9:**
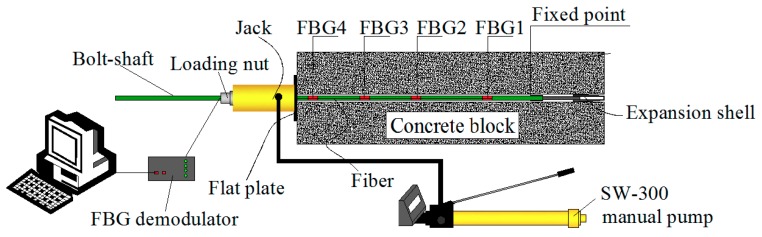
Schematic of the experiment.

**Figure 10:**
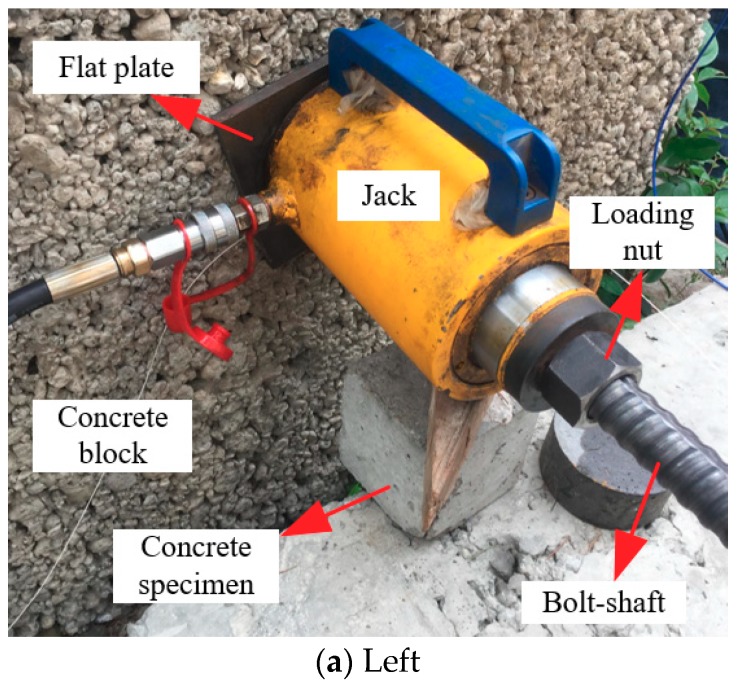

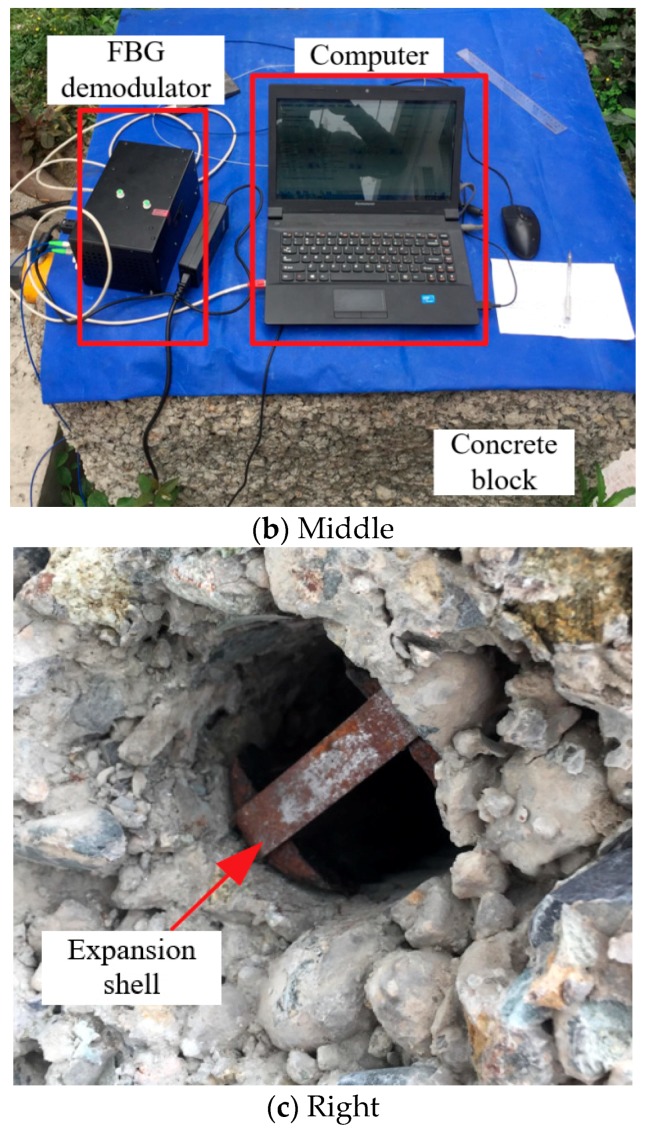
Onsite testing.

**Figure 11:**
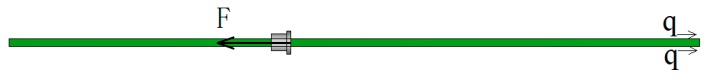
Schematic of rock bolt force.

In the loading mode of the prestressed anchor, named active loading mode, as illustrated in Figure 9, Figure 10 and Figure 11, the expansion shell is used as the fixed component on one side of the concrete block, and the load was directly applied on the bolt-shaft to simulate the force of the prestressed anchor. In the following analysis, this scheme is called “Active”.

**Figure 12:**
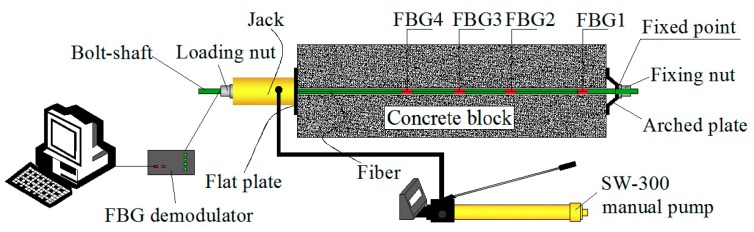
Schematic diagram of the experiment.

**Figure 13:**
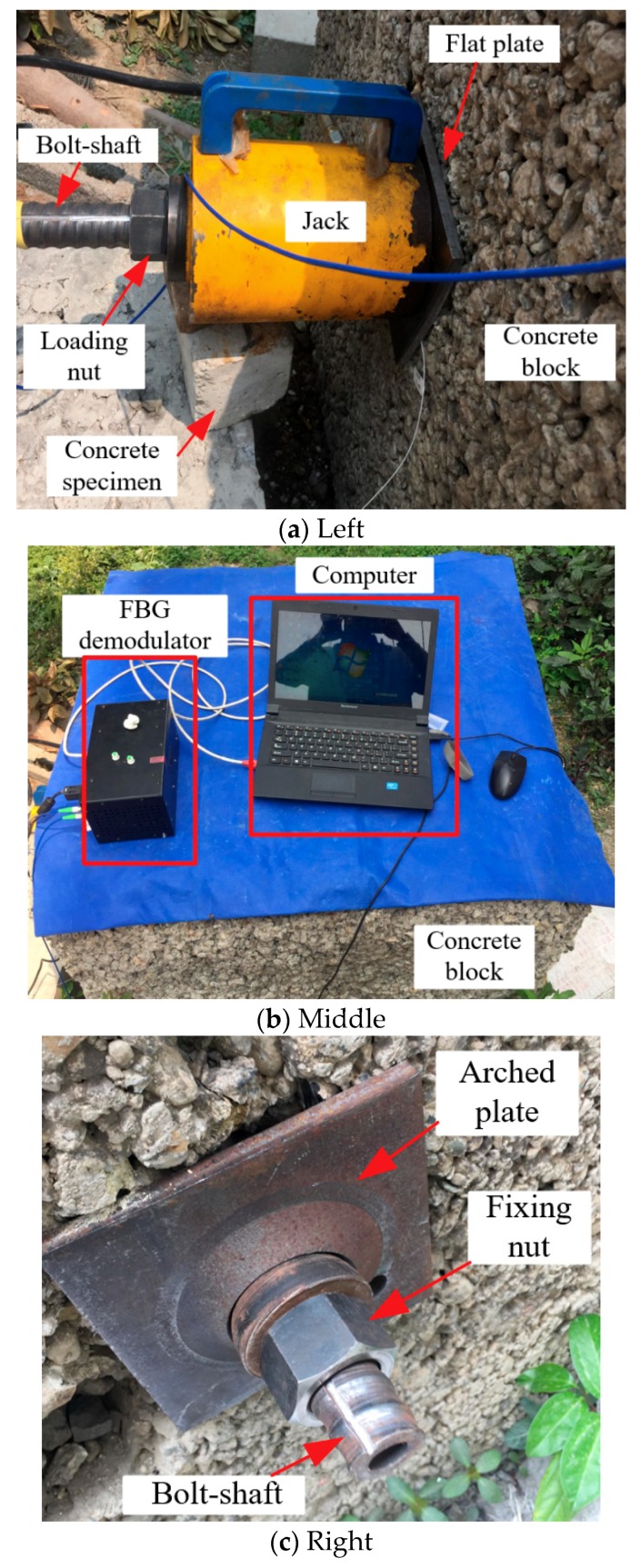
Onsite testing.

**Figure 14:**
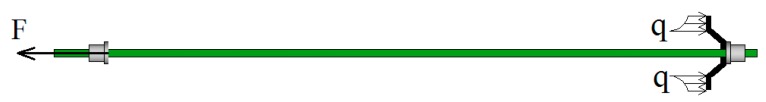
Schematic diagram of rock bolt force.

The loading mode of the non-prestressed anchor, named passive loading mode, is similar to the cement grouted rock bolt system, as illustrated in Figure 12, Figure 13 and Figure 14. The arched plate and nut are used as the fixed component on one side of the concrete block, with the load applied on the other side. Therefore, the concrete block was loaded, and the load would be transferred to the arched plate on the opposite side, and finally transferred to the bolt-shaft to simulate the force of the non-prestressed anchor. In the following analysis, this scheme is called “Passive”.

**Figure 15:**
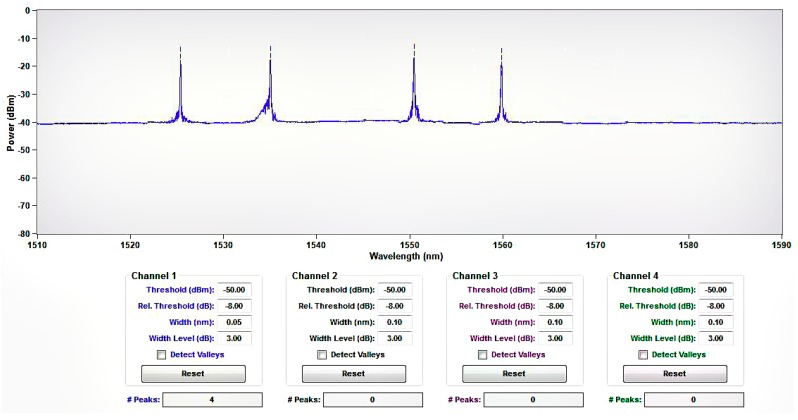
Initial central wavelength distribution of FBG1~FBG4 in Series.

In this test, it takes less than one minute for each stage of the loading process, such as from 5 to 10 kN, and the bolt-shaft was placed in the borehole of a concrete block. Therefore, the wavelength drift caused by the temperature in one stage of loading process is small, and the wavelength drift caused by the temperature change was ignored in the data processing procedure. The center wavelengths and the calibration *K_ε_* values (provided by manufacturers according to the calibration test) of the first to the fourth FBG sensors used in the test were 1524.843, 1534.761, 1550.054, and 1559.731 nm (the central wavelength of adjacent FBG sensors is greater than 9 nm), and 1.1279 ± 0.01, 1.1473 ± 0.01, 1.1602 ± 0.01, and 1.1674 ± 0.01 pm/με, respectively. The relevant information of the FBG sensor used in the test is as follows: the range is ±1500 με, the resolution is 1 με, the fiber type is SMF-28, and the linear correlation coefficient of calibration test data is larger than 99.9%. Figure 15 shows the central wavelength distribution of FBG1~FBG4 under 0 load.

## 4. Experimental Materials and Processes

**Figure 16:**
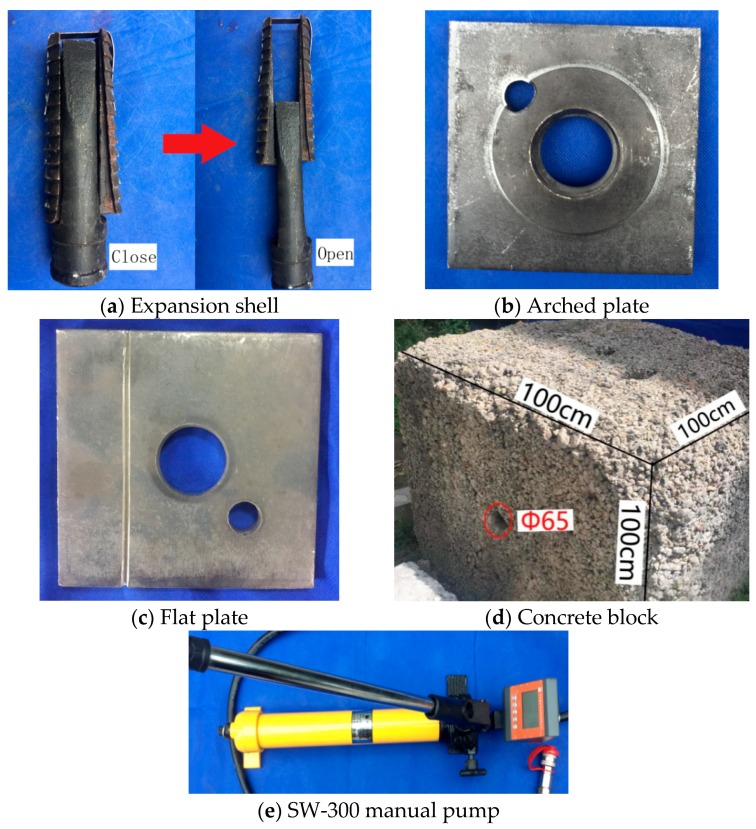
Experimental materials and instruments.

The dimensions of the bolt-shaft used in the test are as follows: the outer diameter is 30 mm, the inner diameter is 17 mm, the wall thickness is 6.5 mm, the cross-sectional area is 480 mm^2^, and the entire length is 1.5 m. The material of the bolt-shaft is Q420 seamless steel tube. The expansion shell used in the test is a push–pull type expansion shell. When the bolt-shaft drives the inner wedge of the expanding shell, the outer shell clip will open, as shown in Figure 16a, and it will then jam the wall of the borehole. The plates used in the test are shown in Figure 16b,c. The dimensions of the arched plate used in the test are as follows: the edge length is 15 cm × 15 cm, the thickness is 1 cm, the arch height is 3 cm, the diameter of the middle arch is 10 cm, and the diameter of the central opening is 5.5 cm. Additionally, the edge length of the flat plate is 15 cm × 15 cm, and its thickness is 1 cm.

A concrete block, as shown in Figure 16d, was used to simulate the environment where the rock bolt would be installed. The concrete block has a dimension of 100 cm × 100 cm × 100 cm and has an opening in the middle and a borehole diameter of 65 mm. The test loading device, as shown in Figure 16e, is an SW-300 manual pump with a maximum pulling force of 300 kN.

The manual pump was used to evenly and slowly apply the load at 5, 10, 15, and 20 kN in “Active” and at 5, 10, 15, 20, 25, 30, 35, and 40 kN in “Passive”. Meanwhile, the wavelength of the FBG sensors integrated with the rock bolt was measured and processed by the interrogator.

## 5. Experimental Results and Analyses

**Figure 17:**
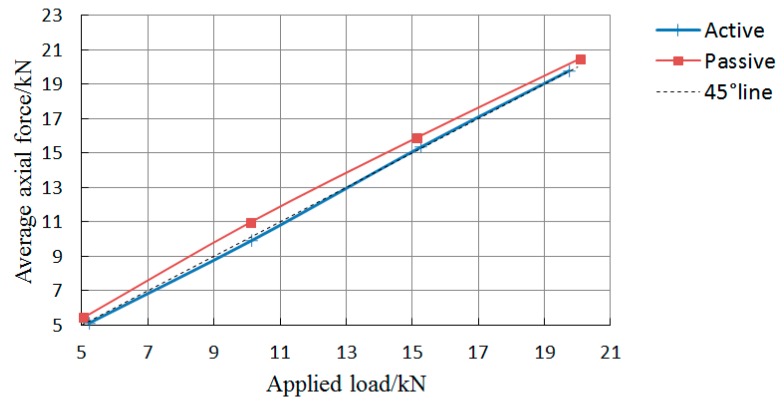
The relationship between the average value of the bolt-shaft and the applied load.

**Figure 18:**
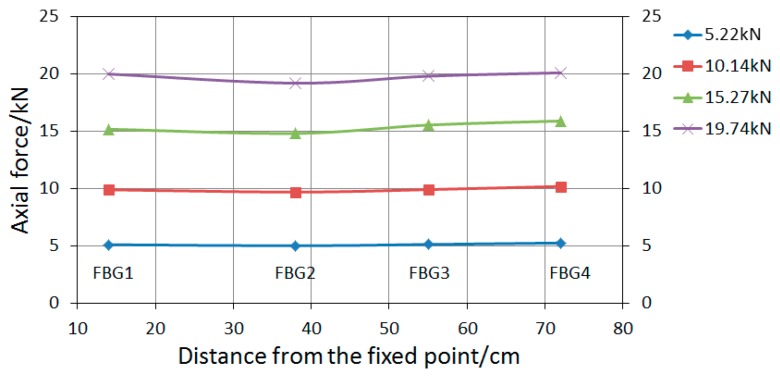
The variation curve of the axial force of the bolt-shaft in “Active”.

Each group of tests was conducted three times. Based on Equation (10), the conversion coefficient between wavelength offset measured by FBG sensors and axis force of bolt-shaft is 100.1, 98.4, 97.3, and 96.7 N/pm. The axial force data (including the average value) of each measuring point are listed in Table 1. The relationship of the average value of the bolt-shaft with the applied load is shown in Figure 16. The relationships of the axial force of the rock bolt with the distance from the fixed point in “Active” and “Passive” are shown in Figure 17 and Figure 18, respectively.

**Figure 19:**
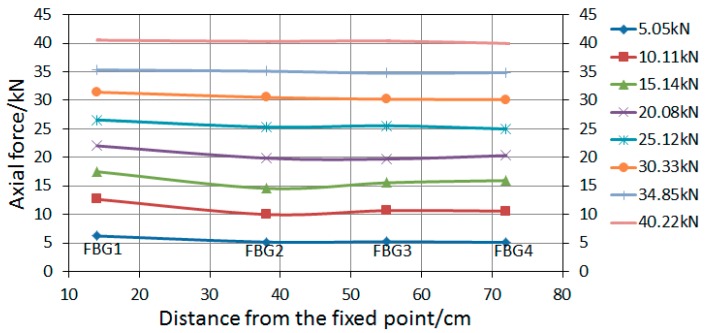
The variation curve of the axial force of the bolt-shaft in “Passive”.

As shown in Figure 17, under the same applied load, the average axial force of the bolt-shaft in “Passive” is slightly larger than that in “Active”, and the maximum difference, 1.08 kN, occurs at the applied load of 10 kN. There is a difference in the average axial force of the bolt-shaft of the above two load conditions, indicating that different loading modes have certain influence on the force of the bolt-shaft. As shown in Figure 18 and Figure 19, in “Active”, the axial force of the bolt-shaft is basically unchanged along the length direction, with the axial force basically equal to the applied load. The force mode of the bolt-shaft is very similar to the two-force bar mode. In “Passive”, the values of axial force at FBG2~FBG4 are basically the same, and it is close to the applied load value. The maximum difference, 0.81 kN, occurs on the FBG4 at the applied load of 15.14 kN. Compared with the applied load, the axial force of FBG1 measurement points changes more obviously. When the applied load is 5.05, 10.11, 15.14, 20.08, 25.12, and 30.33 kN, the corresponding axial force of the FBG1 is 6.23, 12.66, 17.50, 22.0, 26.58, and 31.44 kN. The corresponding differences are 1.18, 2.55, 2.36, 1.92, 1.46, and 1.11 kN, which obviously exceed the maximum difference (0.81 kN) of FBG2~FBG4 under a load between 5 and 30 kN. However, when the applied load is 35 and 40 kN, the corresponding difference (0.50 and 0.33 kN) is less than 0.81 kN. The reason for the difference of the axial force of the bolt-shaft in “Active” and “Passive”, mentioned above, should be caused by different loading modes. In the active loading mode, since the force on the bolt-shaft comes from the loading device, the force mode of the entire bolt-shaft is basically the same as that of the two-force bar model. In the passive loading mode, the reason for the above phenomena is that under the passive loading mode, part of the force of the shaft comes from the plate, and the force exerted by the plate on the bolt-shaft comes from the interaction force between the surrounding rock and the plate. Therefore, when the applied load is small, there is a dynamic adjustment process between the plate and the surrounding rock, which leads to the increase of the force on the bolt-shaft. However, when the value exceeds a certain value, the adjustment process is basically completed. Therefore, the force on the plate tends to be uniform, and the force applied on the bolt-shaft tends to be stable.

**Figure 20:**
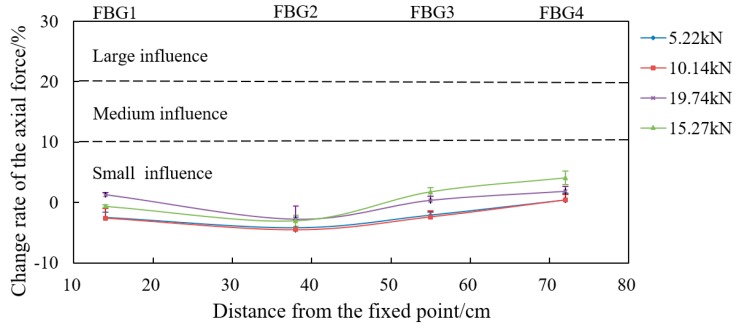
The variation curve of the change rate of the bolt-shaft axial force in “Active”.

**Figure 21:**
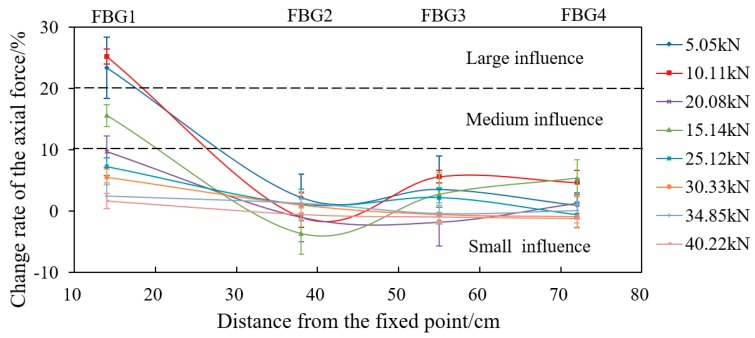
The variation curve of the change rate of the bolt-shaft axial force in “Passive”.

In order to better analyze the influence of loading mode on the force of the bolt-shaft, the relationship between the change rate of axial force (axial force of the FBG-applied load)/applied load) and the distance from the fixed end is plotted based on the load value applied in each loading, as shown in Figure 20 and Figure 21. For the purpose of analysis, it is now defined that the change rate of more than 20% is “large influence”, less than 10% is “small influence”, and 10%~20% as “medium influence” in the following analysis.

As shown in Figure 20, none of the change rates of the bolt-shaft axial force in “Active” are in the area of “large influence” or “medium influence”, and all are located in the “small influence” area. The change rate of the bolt-shaft axial force of each measuring point is basically the same, and there is no significant change with the increase or decrease of the applied load, which clearly shows that the bearing mode of the bolt-shaft is identical to that of the two-force bar model.

As shown in Figure 21, the change rates of the bolt-shaft axial force at FBG2~FBG4 in “Passive” are all located in “small influence” area, which is basically consistent with the change rates of corresponding measuring points in “Active”. However, the change rate of axial force of FBG1 in “Passive” is obviously different from that of FBG2~FBG4 in “Passive” and FBG1 in “Active”. When the applied load is between 5 and 15 kN, the change rate of axial force of FBG1 is basically in the area of “large influence” or “medium influence”. When the applied load is about 20 kN, the change rate of axial force of FBG1 may be in the area of “medium influence” or “small influence”. When the applied load is between 25 and 40 kN, the change rate of axial force of FBG1 is in the area of “small influence”. It also shows that with the increase of applied load, the change rate of axial force of FBG1 tends to decrease. It also indicates that with the increase of the applied load, the influence of the loading mode on the force of the bolt-shaft tends to decrease.

**Figure 22:**
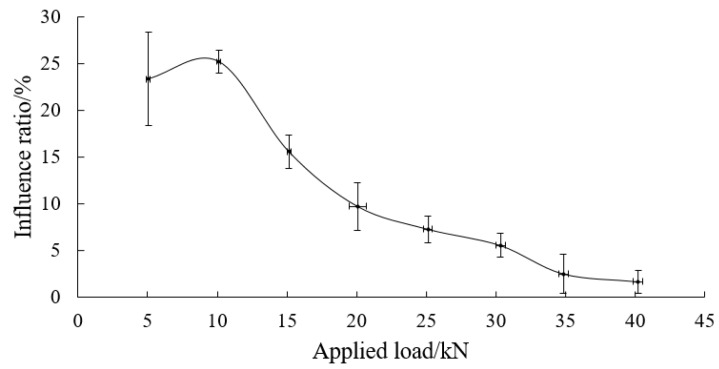
The relation curve between the applied load and the change rate of axial force of FBG1 in “Passive”.

As shown in Figure 22, with the applied load less than 15 kN, the change rate of the axial force on FBG1 is greater than 10%. When the applied load is 20.08 ± 0.63 kN, it is 9.71% ± 2.57% (located in the area of “medium influence” or “small influence”). With the applied load greater than 20 kN, it is less than 10%. At the same time, when the applied load increases, the change rate of the axial force on FBG1 decreases gradually. When the applied load is 34.85 ± 0.35 and 40.22 ± 0.34 kN, it is 2.49 ± 2.06% and 1.6 ± 1.19%, respectively. This shows that the loading mode barely affects the axial force of the bolt-shaft when the applied load exceeds 35 kN.

## 6. Conclusions and Future Work

In this paper, under the background of prestressed anchor and non-prestressed anchor, by using FBG sensing technology, we designed two loading models equivalent to prestressed anchor and non-prestressed anchor in mechanical mechanism. Through the analysis of the experimental data, the following conclusions are obtained:

(1) The obvious differences are mainly obtained on FBG1 in the test, which indicates that different loading modes have limited influence on the axial force of the bolt-shaft.

(2) In the active loading mode, the axial force state of the bolt-shaft is similar to that of the two-force bar.

(3) In the passive support pattern, due to the plate, a certain area surrounding the plate where the axial force of the bolt-shaft is greatly influenced appears. The obvious variation is mainly obtained on FBG1. With the increase of the applied load, the change rate of FBG1 decreases gradually and eventually approaches the inherent error of the measurement system. This shows that with the increase of the load, the influence of the loading modes on the shaft decreases gradually.

Rock bolts are often subjected to corrosion, especially the ones in coastal environment where chloride diffusion accelerates the corrosion [19,40,41]. In future work, we will use the rock bolts integrated with fiber optical sensors to monitor the stress changing along its shaft and to estimate the remaining force bearing capacity of a corroded rock bolt.

## Figures and Tables

**Table 1:** The axial force of the measuring point.

Name		Applied Load/kN	FBG1	FBG2	FBG3	FBG4	Average
	Point Number						
Active	5.22 ± 0.09		5.09 ± 0.05	5.00 ± 0.09	5.11 ± 0.03	5.24 ± 0.06	5.11 ± 0.05
	10.14 ± 0.08		9.88 ± 0.16	9.69 ± 0.16	9.90 ± 0.10	10.18 ± 0.10	9.91 ± 0.13
	15.27 ± 0.14		15.17 ± 0.04	14.81 ± 0.14	15.54 ± 0.11	15.89 ± 0.18	15.35 ± 0.12
	19.74 ± 0.16		19.99 ± 0.08	19.20 ± 0.44	19.81 ± 0.12	20.10 ± 0.17	19.78 ± 0.20
Passive	5.05 ± 0.15		6.23 ± 0.25	5.16 ± 0.24	5.23 ± 0.28	5.10 ± 0.11	5.43 ± 0.22
	10.11 ± 0.12		12.66 ± 0.13	10.01 ± 0.21	10.68 ± 0.10	10.58 ± 0.22	10.98 ± 0.16
	15.14 ± 0.13		17.50 ± 0.27	14.58 ± 0.59	15.55 ± 0.51	15.95 ± 0.46	15.90 ± 0.46
	20.08 ± 0.63		22.03 ± 0.52	19.88 ± 0.87	19.72 ± 0.78	20.34 ± 0.78	20.49 ± 0.74
	25.12 ± 0.32		26.58 ± 0.36	25.33 ± 0.67	25.56 ± 0.39	24.99 ± 0.51	25.62 ± 0.48
	30.33 ± 0.34		31.44 ± 0.39	30.53 ± 0.68	30.22 ± 0.44	30.15 ± 0.57	30.59 ± 0.52
	34.85 ± 0.35		35.35 ± 0.72	35.11 ± 0.23	34.77 ± 0.61	34.87 ± 0.51	35.03 ± 0.51
	40.22 ± 0.34		40.55 ± 0.48	40.11 ± 0.44	40.03 ± 0.31	39.98 ± 0.30	40.17 ± 0.38
